# Poly[μ_5_-{hydrogen bis­[(*E*)-cinnamato]}-caesium]

**DOI:** 10.1107/S1600536814000804

**Published:** 2014-01-18

**Authors:** Graham Smith

**Affiliations:** a Science and Engineering Faculty, Queensland University of Technology, GPO Box 2434, Brisbane, Queensland 4001, Australia

## Abstract

In the structure of the title polymeric complex, [Cs(C_9_H_7_O_2_)(C_9_H_8_O_2_)]_*n*_, a caesium salt of *trans*-cinnamic acid, the Cs^+^ ions of the two individual irregular CsO_8_ coordination polyhedra lie on twofold rotation axes and are linked by four bridging carboxyl O-atom donors from two cinnamate ligand species. These two ligand components are inter­linked through a delocalized H atom within a short O⋯H⋯O hydrogen bond. Structure extension gives a two-dimensional coordination polymer which lies parallel to (001). The structure was determined from a crystal twinned by non-merohedry, with a twin component ratio of approximately 1:1.

## Related literature   

For the structures of the ammonium salts of hydrogen bis­(3-chloro­cinnamate) and hydrogen bis­(3-bromo­cinnamate), see: Chowdhury & Kariuki (2006 ▶). For structures of alkali metal salts of ring-substituted *trans*-cinnamic acid, see: Kariuki *et al.* (1994 ▶, 1995 ▶); Crowther *et al.* (2008 ▶); Smith & Wermuth (2009 ▶, 2011 ▶). For the structure of *trans*-cinnamic acid, see: Wierda *et al.* (1989 ▶); Abdelmoty *et al.* (2005 ▶).
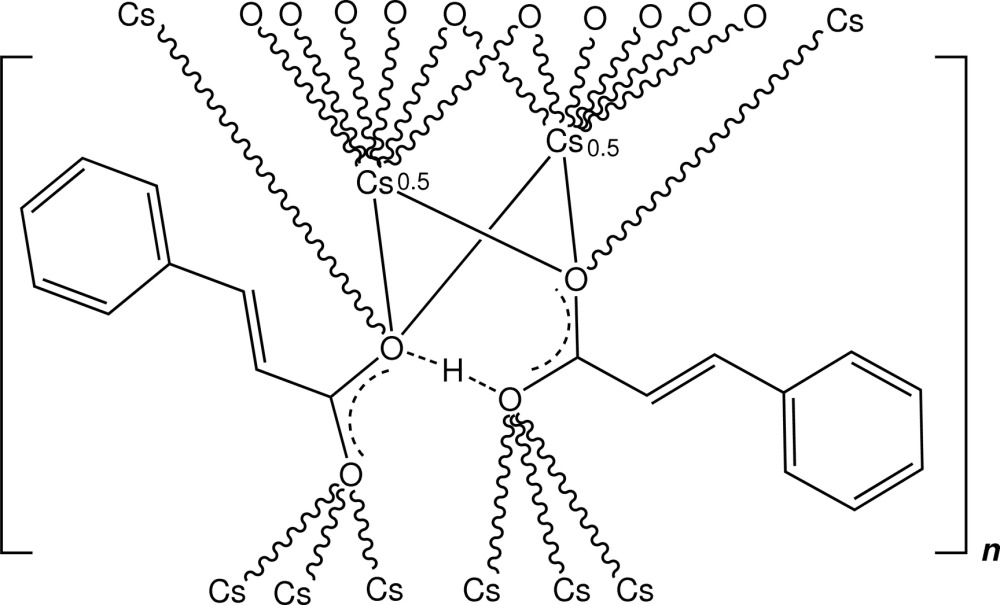


## Experimental   

### 

#### Crystal data  

[Cs(C_9_H_7_O_2_)(C_9_H_8_O_2_)]
*M* *_r_* = 428.21
Monoclinic, 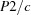

*a* = 7.8608 (6) Å
*b* = 5.6985 (7) Å
*c* = 38.817 (3) Å
β = 98.733 (6)°
*V* = 1718.6 (3) Å^3^
*Z* = 4
Mo *K*α radiation
μ = 2.17 mm^−1^
*T* = 200 K
0.35 × 0.35 × 0.06 mm

#### Data collection  

Oxford Diffraction Gemini-S CCD-detector diffractometer
Absorption correction: multi-scan (*CrysAlis PRO*; Agilent, 2013 ▶) *T*_min_ = 0.711, *T*_max_ = 0.980
6675 measured reflections
3353 independent reflections
2552 reflections with *I* > 2σ(*I*)
*R*_int_ = 0.046

#### Refinement  

*R*[*F* ^2^ > 2σ(*F* ^2^)] = 0.071
*wR*(*F* ^2^) = 0.144
*S* = 1.19
3353 reflections
210 parameters
H-atom parameters constrained
Δρ_max_ = 1.26 e Å^−3^
Δρ_min_ = −2.19 e Å^−3^

### 

Data collection: *CrysAlis PRO* (Agilent, 2013 ▶); cell refinement: *CrysAlis PRO*; data reduction: *CrysAlis PRO*; program(s) used to solve structure: *SHELXS97* (Sheldrick, 2008 ▶); program(s) used to refine structure: *SHELXL97* (Sheldrick, 2008 ▶) within *WinGX* (Farrugia, 2012 ▶); molecular graphics: *PLATON* (Spek, 2009 ▶); software used to prepare material for publication: *PLATON*.

## Supplementary Material

Crystal structure: contains datablock(s) global, I. DOI: 10.1107/S1600536814000804/wm2798sup1.cif

Structure factors: contains datablock(s) I. DOI: 10.1107/S1600536814000804/wm2798Isup2.hkl

Supporting information file. DOI: 10.1107/S1600536814000804/wm2798Isup3.cml

CCDC reference:

Additional supporting information:  crystallographic information; 3D view; checkCIF report

## Figures and Tables

**Table 1:** Selected bond lengths (Å)

Cs1—O13*B*	3.060 (8)
Cs1—O14*A*	3.182 (8)
Cs1—O13*A* ^i^	3.132 (9)
Cs1—O14*B* ^i^	3.183 (9)
Cs1—O13*B* ^ii^	3.060 (8)
Cs1—O14*A* ^ii^	3.182 (8)
Cs1—O13*A* ^iii^	3.132 (9)
Cs1—O14*B* ^iii^	3.183 (9)
Cs2—O13*B*	3.063 (8)
Cs2—O14*A*	3.377 (9)
Cs2—O13*A* ^i^	3.108 (9)
Cs2—O14*B* ^i^	3.130 (9)
Cs2—O13*B* ^iv^	3.063 (8)
Cs2—O14*A* ^iv^	3.377 (9)
Cs2—O13*A* ^v^	3.108 (9)
Cs2—O14*B* ^v^	3.130 (9)

Symmetry codes: (i) 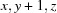; (ii) 
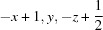
; (iii) 
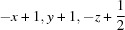
; (iv) 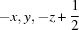; (v) 
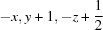
.

**Table 2:** Hydrogen-bond geometry (Å, °)

*D*—H⋯*A*	*D*—H	H⋯*A*	*D*⋯*A*	*D*—H⋯*A*
O14*B*—H14*B*⋯O14*A*	1.21	1.25	2.462 (10)	180

**Fig. 1.**
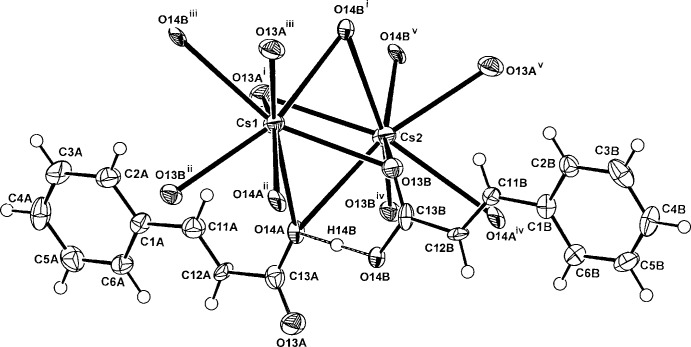
The atom-numbering scheme and the molecular configuration of the two ligands and the two CsO8 coordination polyhedra of the title complex, with non-H atoms drawn with displacement ellipsoids at the 40% probability level. The two Cs+ cations lie on twofold rotation axes. The O14A···O14B hydrogen bond with the delocalized H atom (H14B) is shown as a dashed link. [For symmetry codes: see Table 1].

**Fig. 2.**
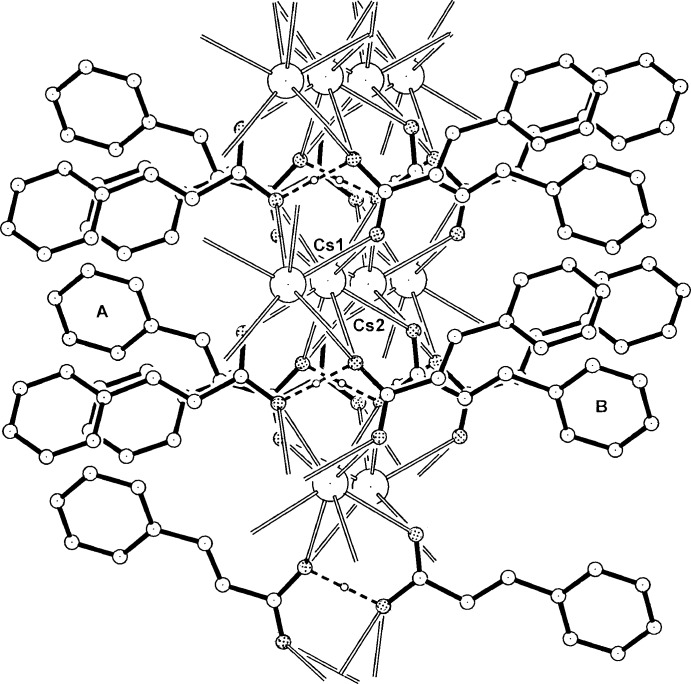
A view of the partially expanded polymeric extension of the structure viewed along the approximate a-cell direction. C-bound H atoms are omitted. A and B denote the two different ligand components.

**Fig. 3.**
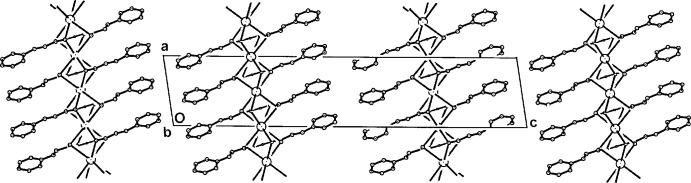
The packing of the layered structure of compound (I) viewed along b.

## supplementary crystallographic information

### 1. Comment

The crystal structure of *trans*-cinnamic acid was reported by Wierda *et al.* (1989) and Abdelmoty *et al.* (2005). The alkali metal salts of *trans*-cinnamic acid are unknown in the crystallographic literature although a limited number of examples of salts of ring-substituted cinnamates have been reported, *e.g.* the sodium salts of 2-nitrocinnamate [a dihydrate (Smith & Wermuth, 2009)], of 2-chlorocinnamate [a dihydrate (Kariuki *et al.*, 1995)], of 3-chlorocinnamate [anhydrous (Crowther *et al.*, 2008), of 4-chlorocinnamate [a dihydrate (Kariuki *et al.*, 1994); potassium salts of 3-chloro- and 3-bromocinnamate [both anhydrous (Crowther *et al.*, 2008)]; and a rubidium salt of 2-nitrocinnamate [a monohydrate (Smith & Wermuth, 2011)].

The reaction of *trans*-cinnamic acid with caesium hydroxide in aqueous ethanol afforded crystals of the title complex, [Cs(C_9_H_7_O_2_)(C_9_H_8_O_2_)]_n_, (I), the structure of which is reported herein.

In the structure of (I) the asymmetric unit (Fig. 1) comprises two independent irregular CsO_8_ coordination polyhedra [Cs1—O, 3.060 (8)–3.183 (9) Å; Cs2—O, 3.063 (9)–3.377 (9) Å: Table 1], in which the Cs^+^ ions lie on a twofold rotation axis and are linked by four bridging carboxyl O-donors from the two *trans*-cinnamate ligand species. These two ligand species are inter-linked through a delocalized H atom on an approximately central intermediate site within a short O14*A*···H14*B*··· O14*B* hydrogen bond [2.462 (10) Å] (Table 2). Although this phenomenon involving coordinating dimeric carboxylate species is not known among the alkali metal substituted-cinnamate structures, it is found in both ammonium hydrogen bis(3-chlorocinnamate) and ammonium hydrogen bis(3-bromocinnamate) (Chowdhury & Kariuki, 2006), with the O···H···O values [2.554 (6) Å for the 3-Cl-analogue and 2.466 (5) Å for the 3-Br-analogue] similar to that in the structure of (I). In this complex, the two Cs^+^ ions are quadruply bridged giving a Cs1···Cs2 separation of 3.9318 (3) Å and generate an overall two-dimensional coordination polymer lying parallel to (001) (Figs. 2, 3). No inter-ring π–π interactions are present in the structure [minimun ring centroid separation = 4.826 (8) Å].

The two linked cinnamate species in the title complex are close to coplanar [inter-ring dihedral angle = 3.9 (6)°], with the side chain carboxyl group of the *A* ligand component slightly rotated out of the plane [torsion angle C11*A*—C12*A*— C13*A*—O13*A* = 169.0 (13)°] compared to that of the *B* ligand component [torsion angle C11*B*—C12*B*— C13*B*—O14*B* = -179.2 (11)°]. With the analogous ammonium hydrogen salts of the 3-chloro- and 3-bromocinnamates (Chowdhury & Kariuki, 2006), the two cinnamate components are related either by crystallographic inversion symmetry (3-Cl) with the two benzene rings essentially planar, or by twofold rotational symmetry (3-Br) with the two rings significantly rotated out of the least-squares plane [inter-ring dihedral angle = 29.8 (2)°].

### 2. Experimental

The title compound was synthesized by heating together for 10 minutes, 148 mg (1.0 mmol) of *trans*-cinnamic acid and 75 mg (0.5 mmol) of CsOH in 15 ml of an 1:9 (vol/vol) ethanol–water mixture. Partial room temperature evaporation of the solution gave colourless elongated crystals of the title complex from which a specimen was cleaved for the X-ray analysis. These crystals were invariably twinned, a feature identified in the later structure solution and refinement routines.

### 3. Refinement

Hydrogen atoms were placed in calculated positions [C—H = 0.95 Å] and allowed to ride in the refinement, with *U*_iso_(H) = 1.2*U*_eq_(C). The carboxylic acid H-atom was found to be delocalized in a site approximating to midway between two carboxyl O-atoms of the dimeric acid–anion unit and was subsequently allowed to ride at that site, with *U*_iso_(H) = 1.5*U*_eq_(O). The presence of a non-merohedral twin was identified using TwinRotMat within *PLATON* (Spek, 2009) (twin law: 1 0 0, 0 1 0, 1.5 0 1) reducing the conventional *R*-factor from 0.23 to 0.072, with a final BASF factor (HKLF 5 format) of 0.4836. Maximum and minimum residual electron densities were 1.26 eÅ^-3^ (1.00 Å from Cs1) and -2.19 eÅ^-3^ (1.94 Å from H14*B*), respectively.

### Crystal data

**Table:**

[Cs(C_9_H_7_O_2_)(C_9_H_8_O_2_)]	*F*(000) = 840
*M**_r_* = 428.21	*D*_x_ = 1.655 Mg m^−^^3^
Monoclinic, *P*2/*c*	Mo *K*α radiation, λ = 0.71073 Å
Hall symbol: -P 2yc	Cell parameters from 1674 reflections
*a* = 7.8608 (6) Å	θ = 3.6–28.2°
*b* = 5.6985 (7) Å	µ = 2.17 mm^−^^1^
*c* = 38.817 (3) Å	*T* = 200 K
β = 98.733 (6)°	Plate, colourless
*V* = 1718.6 (3) Å^3^	0.35 × 0.35 × 0.06 mm
*Z* = 4	

### Data collection

**Table:**

Oxford Diffraction Gemini-S CCD-detector diffractometer	3353 independent reflections
Radiation source: Enhance (Mo) X-ray source	2552 reflections with *I* > 2σ(*I*)
Graphite monochromator	*R*_int_ = 0.046
Detector resolution: 16.077 pixels mm^-1^	θ_max_ = 26.0°, θ_min_ = 3.2°
ω scans	*h* = −9→9
Absorption correction: multi-scan (*CrysAlis PRO*; Agilent, 2013)	*k* = −7→7
*T*_min_ = 0.711, *T*_max_ = 0.980	*l* = −11→47
6675 measured reflections	

### Refinement

**Table:**

Refinement on *F*^2^	Primary atom site location: structure-invariant direct methods
Least-squares matrix: full	Secondary atom site location: difference Fourier map
*R*[*F*^2^ > 2σ(*F*^2^)] = 0.071	Hydrogen site location: inferred from neighbouring sites
*wR*(*F*^2^) = 0.144	H-atom parameters constrained
*S* = 1.19	*w* = 1/[σ^2^(*F*_o_^2^) + (0.020*P*)^2^ + 18.34*P*] where *P* = (*F*_o_^2^ + 2*F*_c_^2^)/3
3353 reflections	(Δ/σ)_max_ = 0.001
210 parameters	Δρ_max_ = 1.26 e Å^−^^3^
0 restraints	Δρ_min_ = −2.19 e Å^−^^3^

### Special details

**Table:**

Geometry. Bond distances, angles *etc*. have been calculated using the rounded fractional coordinates. All su's are estimated from the variances of the (full) variance-covariance matrix. The cell e.s.d.'s are taken into account in the estimation of distances, angles and torsion angles
Refinement. Refinement of *F*^2^ against ALL reflections. The weighted *R*-factor *wR* and goodness of fit *S* are based on *F*^2^, conventional *R*-factors *R* are based on *F*, with *F* set to zero for negative *F*^2^. The threshold expression of *F*^2^ > σ(*F*^2^) is used only for calculating *R*-factors(gt) *etc*. and is not relevant to the choice of reflections for refinement. *R*-factors based on *F*^2^ are statistically about twice as large as those based on *F*, and *R*- factors based on ALL data will be even larger.

### Fractional atomic coordinates and isotropic or equivalent isotropic displacement parameters (Å^2^)

**Table:**

	*x*	*y*	*z*	*U*_iso_*/*U*_eq_	
Cs1	0.50000	0.6438 (2)	0.25000	0.0261 (3)	
Cs2	0.00000	0.6257 (2)	0.25000	0.0300 (3)	
O13A	0.2828 (13)	−0.1322 (15)	0.3028 (2)	0.037 (3)	
O13B	0.2180 (12)	0.3903 (14)	0.20079 (19)	0.030 (3)	
O14A	0.3036 (12)	0.2355 (14)	0.28374 (18)	0.029 (3)	
O14B	0.2197 (13)	0.0396 (14)	0.22718 (18)	0.033 (3)	
C1A	0.5037 (15)	0.4316 (19)	0.3905 (3)	0.025 (3)	
C1B	0.0000 (15)	0.079 (2)	0.1031 (3)	0.025 (3)	
C2A	0.5963 (19)	0.636 (2)	0.3964 (3)	0.035 (4)	
C2B	0.0132 (16)	0.208 (2)	0.0728 (3)	0.028 (3)	
C3A	0.6620 (19)	0.707 (2)	0.4301 (4)	0.042 (5)	
C3B	−0.0664 (16)	0.128 (3)	0.0403 (3)	0.040 (4)	
C4A	0.6346 (18)	0.574 (3)	0.4585 (3)	0.042 (5)	
C4B	−0.1524 (18)	−0.082 (2)	0.0373 (3)	0.041 (4)	
C5A	0.5426 (19)	0.373 (3)	0.4529 (3)	0.039 (4)	
C5B	−0.1628 (16)	−0.210 (2)	0.0668 (3)	0.035 (4)	
C6A	0.4764 (15)	0.299 (2)	0.4191 (3)	0.029 (4)	
C6B	−0.0870 (15)	−0.133 (2)	0.0997 (3)	0.031 (4)	
C11A	0.4356 (17)	0.359 (2)	0.3541 (3)	0.032 (4)	
C11B	0.0831 (14)	0.173 (2)	0.1369 (3)	0.025 (3)	
C12A	0.3722 (13)	0.155 (2)	0.3444 (3)	0.023 (3)	
C12B	0.1178 (15)	0.054 (2)	0.1672 (3)	0.026 (3)	
C13A	0.3148 (16)	0.076 (2)	0.3078 (3)	0.028 (4)	
C13B	0.1901 (15)	0.176 (2)	0.1998 (3)	0.027 (4)	
H2A	0.61560	0.73090	0.37720	0.0410*	
H2B	0.07640	0.35110	0.07440	0.0340*	
H3A	0.72650	0.84820	0.43360	0.0510*	
H3B	−0.06090	0.21990	0.02000	0.0480*	
H4A	0.67930	0.62310	0.48150	0.0500*	
H4B	−0.20410	−0.13800	0.01520	0.0490*	
H5A	0.52280	0.28000	0.47230	0.0470*	
H5B	−0.22320	−0.35490	0.06480	0.0410*	
H6A	0.41240	0.15700	0.41580	0.0350*	
H6B	−0.09500	−0.22560	0.11970	0.0370*	
H11A	0.43910	0.47280	0.33640	0.0380*	
H11B	0.11540	0.33430	0.13740	0.0290*	
H12A	0.36110	0.04540	0.36230	0.0280*	
H12B	0.09570	−0.10990	0.16760	0.0320*	
H14B	0.26080	0.13620	0.25500	0.0500*	

### Atomic displacement parameters (Å^2^)

**Table:**

	*U*^11^	*U*^22^	*U*^33^	*U*^12^	*U*^13^	*U*^23^
Cs1	0.0221 (5)	0.0212 (5)	0.0350 (5)	0.0000	0.0041 (5)	0.0000
Cs2	0.0232 (5)	0.0238 (6)	0.0438 (6)	0.0000	0.0075 (6)	0.0000
O13A	0.056 (5)	0.027 (5)	0.028 (4)	−0.001 (6)	0.003 (4)	−0.006 (4)
O13B	0.047 (5)	0.026 (4)	0.016 (4)	−0.004 (5)	0.000 (4)	0.000 (3)
O14A	0.046 (6)	0.033 (5)	0.006 (3)	−0.003 (4)	−0.006 (3)	−0.005 (3)
O14B	0.055 (5)	0.025 (4)	0.013 (4)	−0.004 (5)	−0.015 (4)	−0.005 (3)
C1A	0.022 (6)	0.022 (6)	0.030 (6)	0.003 (5)	0.006 (5)	−0.009 (5)
C1B	0.020 (5)	0.034 (6)	0.022 (5)	0.004 (6)	0.005 (5)	−0.007 (5)
C2A	0.038 (7)	0.027 (6)	0.039 (6)	−0.004 (7)	0.007 (6)	0.003 (6)
C2B	0.027 (6)	0.028 (6)	0.027 (6)	0.007 (6)	0.000 (5)	−0.003 (5)
C3A	0.044 (9)	0.021 (7)	0.059 (9)	0.012 (6)	−0.002 (7)	−0.024 (6)
C3B	0.043 (8)	0.050 (8)	0.027 (6)	0.026 (8)	0.005 (5)	0.000 (7)
C4A	0.031 (7)	0.059 (10)	0.033 (7)	0.006 (8)	−0.001 (6)	−0.022 (7)
C4B	0.037 (7)	0.053 (8)	0.030 (7)	−0.011 (8)	−0.003 (6)	−0.012 (6)
C5A	0.035 (7)	0.048 (7)	0.035 (6)	−0.003 (8)	0.005 (6)	0.012 (7)
C5B	0.029 (7)	0.021 (6)	0.054 (8)	−0.005 (6)	0.006 (6)	−0.004 (6)
C6A	0.031 (7)	0.027 (7)	0.030 (6)	−0.007 (6)	0.005 (5)	0.004 (5)
C6B	0.032 (7)	0.027 (6)	0.032 (6)	−0.002 (6)	0.002 (5)	−0.002 (6)
C11A	0.035 (7)	0.029 (6)	0.032 (6)	−0.002 (7)	0.006 (6)	0.002 (6)
C11B	0.030 (6)	0.021 (6)	0.021 (5)	−0.004 (5)	−0.001 (4)	0.001 (5)
C12A	0.036 (6)	0.017 (6)	0.016 (5)	−0.008 (5)	0.002 (4)	−0.008 (5)
C12B	0.038 (7)	0.013 (5)	0.027 (6)	−0.009 (5)	0.001 (5)	0.006 (5)
C13A	0.021 (6)	0.036 (8)	0.027 (6)	−0.002 (6)	0.003 (5)	0.002 (5)
C13B	0.023 (6)	0.043 (8)	0.015 (5)	0.000 (6)	−0.001 (5)	−0.010 (6)

### Geometric parameters (Å, º)

**Table:**

Cs1—O13B	3.060 (8)	C2A—C3A	1.392 (19)
Cs1—O14A	3.182 (8)	C2B—C3B	1.397 (17)
Cs1—O13A^i^	3.132 (9)	C3A—C4A	1.38 (2)
Cs1—O14B^i^	3.183 (9)	C3B—C4B	1.37 (2)
Cs1—O13B^ii^	3.060 (8)	C4A—C5A	1.35 (2)
Cs1—O14A^ii^	3.182 (8)	C4B—C5B	1.371 (16)
Cs1—O13A^iii^	3.132 (9)	C5A—C6A	1.401 (17)
Cs1—O14B^iii^	3.183 (9)	C5B—C6B	1.396 (16)
Cs2—O13B	3.063 (8)	C11A—C12A	1.298 (16)
Cs2—O14A	3.377 (9)	C11B—C12B	1.349 (16)
Cs2—O13A^i^	3.108 (9)	C12A—C13A	1.493 (16)
Cs2—O14B^i^	3.130 (9)	C12B—C13B	1.480 (16)
Cs2—O13B^iv^	3.063 (8)	C2A—H2A	0.9500
Cs2—O14A^iv^	3.377 (9)	C2B—H2B	0.9500
Cs2—O13A^v^	3.108 (9)	C3A—H3A	0.9500
Cs2—O14B^v^	3.130 (9)	C3B—H3B	0.9500
O13A—C13A	1.222 (14)	C4A—H4A	0.9500
O13B—C13B	1.240 (14)	C4B—H4B	0.9500
O14A—C13A	1.297 (14)	C5A—H5A	0.9500
O14B—C13B	1.309 (14)	C5B—H5B	0.9500
O14B—H14B	1.2100	C6A—H6A	0.9500
C1A—C11A	1.492 (16)	C6B—H6B	0.9500
C1A—C2A	1.374 (17)	C11A—H11A	0.9500
C1A—C6A	1.386 (16)	C11B—H11B	0.9500
C1B—C11B	1.475 (16)	C12A—H12A	0.9500
C1B—C6B	1.385 (16)	C12B—H12B	0.9500
C1B—C2B	1.404 (16)		
			
O13B—Cs1—O14A	64.0 (2)	Cs2—O14A—C13A	135.0 (8)
O13A^i^—Cs1—O13B	100.7 (2)	Cs1^vi^—O14B—C13B	132.6 (7)
O13B—Cs1—O14B^i^	75.9 (2)	Cs2^vi^—O14B—C13B	129.8 (7)
O13B—Cs1—O13B^ii^	123.7 (2)	Cs1^vi^—O14B—Cs2^vi^	77.04 (18)
O13B—Cs1—O14A^ii^	75.4 (2)	Cs1—O14A—H14B	93.00
O13A^iii^—Cs1—O13B	101.5 (2)	Cs2—O14A—H14B	83.00
O13B—Cs1—O14B^iii^	155.98 (19)	Cs2^vi^—O14B—H14B	100.00
O13A^i^—Cs1—O14A	71.5 (2)	Cs1^vi^—O14B—H14B	90.00
O14A—Cs1—O14B^i^	105.9 (2)	C13B—O14B—H14B	116.00
O13B^ii^—Cs1—O14A	75.4 (2)	C6A—C1A—C11A	122.0 (10)
O14A—Cs1—O14A^ii^	86.0 (2)	C2A—C1A—C6A	118.1 (11)
O13A^iii^—Cs1—O14A	156.1 (2)	C2A—C1A—C11A	119.9 (10)
O14A—Cs1—O14B^iii^	139.65 (18)	C2B—C1B—C6B	118.4 (11)
O13A^i^—Cs1—O14B^i^	58.0 (2)	C2B—C1B—C11B	118.4 (10)
O13A^i^—Cs1—O13B^ii^	101.5 (2)	C6B—C1B—C11B	123.2 (10)
O13A^i^—Cs1—O14A^ii^	156.1 (2)	C1A—C2A—C3A	121.0 (11)
O13A^i^—Cs1—O13A^iii^	131.9 (2)	C1B—C2B—C3B	120.4 (12)
O13A^i^—Cs1—O14B^iii^	87.3 (2)	C2A—C3A—C4A	120.7 (12)
O13B^ii^—Cs1—O14B^i^	155.98 (19)	C2B—C3B—C4B	120.6 (12)
O14A^ii^—Cs1—O14B^i^	139.65 (18)	C3A—C4A—C5A	118.7 (12)
O13A^iii^—Cs1—O14B^i^	87.3 (2)	C3B—C4B—C5B	119.0 (11)
O14B^i^—Cs1—O14B^iii^	89.8 (2)	C4A—C5A—C6A	121.2 (12)
O13B^ii^—Cs1—O14A^ii^	64.0 (2)	C4B—C5B—C6B	121.7 (11)
O13A^iii^—Cs1—O13B^ii^	100.7 (2)	C1A—C6A—C5A	120.4 (11)
O13B^ii^—Cs1—O14B^iii^	75.9 (2)	C1B—C6B—C5B	119.8 (11)
O13A^iii^—Cs1—O14A^ii^	71.5 (2)	C1A—C11A—C12A	126.2 (11)
O14A^ii^—Cs1—O14B^iii^	105.9 (2)	C1B—C11B—C12B	126.6 (11)
O13A^iii^—Cs1—O14B^iii^	58.0 (2)	C11A—C12A—C13A	126.4 (11)
O13B—Cs2—O14A	61.58 (19)	C11B—C12B—C13B	120.7 (10)
O13A^i^—Cs2—O13B	101.2 (2)	O13A—C13A—C12A	118.0 (10)
O13B—Cs2—O14B^i^	76.6 (2)	O13A—C13A—O14A	125.2 (11)
O13B—Cs2—O13B^iv^	128.1 (2)	O14A—C13A—C12A	116.9 (10)
O13B—Cs2—O14A^iv^	84.2 (2)	O14B—C13B—C12B	114.4 (10)
O13A^v^—Cs2—O13B	101.3 (2)	O13B—C13B—O14B	123.4 (10)
O13B—Cs2—O14B^v^	153.1 (2)	O13B—C13B—C12B	122.2 (10)
O13A^i^—Cs2—O14A	69.2 (2)	C1A—C2A—H2A	120.00
O14A—Cs2—O14B^i^	102.6 (2)	C3A—C2A—H2A	119.00
O13B^iv^—Cs2—O14A	84.2 (2)	C1B—C2B—H2B	120.00
O14A—Cs2—O14A^iv^	97.7 (2)	C3B—C2B—H2B	120.00
O13A^v^—Cs2—O14A	160.1 (2)	C2A—C3A—H3A	120.00
O14A—Cs2—O14B^v^	140.74 (18)	C4A—C3A—H3A	120.00
O13A^i^—Cs2—O14B^i^	58.8 (2)	C2B—C3B—H3B	120.00
O13A^i^—Cs2—O13B^iv^	101.3 (2)	C4B—C3B—H3B	120.00
O13A^i^—Cs2—O14A^iv^	160.1 (2)	C3A—C4A—H4A	121.00
O13A^i^—Cs2—O13A^v^	127.3 (2)	C5A—C4A—H4A	121.00
O13A^i^—Cs2—O14B^v^	81.3 (2)	C3B—C4B—H4B	121.00
O13B^iv^—Cs2—O14B^i^	153.1 (2)	C5B—C4B—H4B	120.00
O14A^iv^—Cs2—O14B^i^	140.74 (18)	C4A—C5A—H5A	119.00
O13A^v^—Cs2—O14B^i^	81.3 (2)	C6A—C5A—H5A	119.00
O14B^i^—Cs2—O14B^v^	82.2 (2)	C4B—C5B—H5B	119.00
O13B^iv^—Cs2—O14A^iv^	61.58 (19)	C6B—C5B—H5B	119.00
O13A^v^—Cs2—O13B^iv^	101.2 (2)	C1A—C6A—H6A	120.00
O13B^iv^—Cs2—O14B^v^	76.6 (2)	C5A—C6A—H6A	120.00
O13A^v^—Cs2—O14A^iv^	69.2 (2)	C1B—C6B—H6B	120.00
O14A^iv^—Cs2—O14B^v^	102.6 (2)	C5B—C6B—H6B	120.00
O13A^v^—Cs2—O14B^v^	58.8 (2)	C1A—C11A—H11A	117.00
Cs1^vi^—O13A—C13A	112.3 (8)	C12A—C11A—H11A	117.00
Cs2^vi^—O13A—C13A	130.2 (8)	C1B—C11B—H11B	117.00
Cs1^vi^—O13A—Cs2^vi^	78.11 (18)	C12B—C11B—H11B	117.00
Cs1—O13B—Cs2	79.91 (18)	C11A—C12A—H12A	117.00
Cs1—O13B—C13B	126.3 (7)	C13A—C12A—H12A	117.00
Cs2—O13B—C13B	109.7 (7)	C11B—C12B—H12B	120.00
Cs1—O14A—Cs2	73.59 (16)	C13B—C12B—H12B	120.00
Cs1—O14A—C13A	145.6 (8)		
			
O14A—Cs1—O13B—Cs2	65.6 (2)	O13B^iv^—Cs2—O14A—Cs1	−160.72 (18)
O14A—Cs1—O13B—C13B	−41.6 (9)	O13B^iv^—Cs2—O14A—C13A	−3.5 (9)
O13A^i^—Cs1—O13B—Cs2	2.5 (2)	O14A^iv^—Cs2—O14A—Cs1	139.03 (16)
O13A^i^—Cs1—O13B—C13B	−104.7 (9)	O14A^iv^—Cs2—O14A—C13A	−63.8 (10)
O14B^i^—Cs1—O13B—Cs2	−50.38 (19)	O14B^v^—Cs2—O14A—Cs1	−100.2 (3)
O14B^i^—Cs1—O13B—C13B	−157.5 (9)	O14B^v^—Cs2—O14A—C13A	57.0 (11)
O13B^ii^—Cs1—O13B—Cs2	114.2 (2)	O13B—Cs2—O13A^i^—Cs1	2.5 (2)
O13B^ii^—Cs1—O13B—C13B	7.0 (10)	O14A—Cs2—O13A^i^—Cs1	56.01 (18)
O14A^ii^—Cs1—O13B—Cs2	158.3 (2)	O13B—Cs2—O14B^i^—Cs1	−49.32 (18)
O14A^ii^—Cs1—O13B—C13B	51.2 (9)	O14A—Cs2—O14B^i^—Cs1	7.01 (18)
O13A^iii^—Cs1—O13B—Cs2	−134.6 (2)	Cs1^vi^—O13A—C13A—O14A	56.2 (15)
O13A^iii^—Cs1—O13B—C13B	118.3 (9)	Cs1^vi^—O13A—C13A—C12A	−123.5 (9)
O14B^iii^—Cs1—O13B—Cs2	−105.2 (5)	Cs2^vi^—O13A—C13A—O14A	−37.0 (18)
O14B^iii^—Cs1—O13B—C13B	147.6 (9)	Cs2^vi^—O13A—C13A—C12A	143.3 (8)
O13B—Cs1—O14A—Cs2	−57.9 (2)	Cs1—O13B—C13B—O14B	31.2 (16)
O13B—Cs1—O14A—C13A	151.0 (13)	Cs1—O13B—C13B—C12B	−149.6 (8)
O13A^i^—Cs1—O14A—Cs2	54.52 (19)	Cs2—O13B—C13B—O14B	−60.7 (13)
O13A^i^—Cs1—O14A—C13A	−96.5 (12)	Cs2—O13B—C13B—C12B	118.6 (10)
O14B^i^—Cs1—O14A—Cs2	7.10 (18)	Cs1—O14A—C13A—O13A	−125.8 (12)
O14B^i^—Cs1—O14A—C13A	−143.9 (12)	Cs1—O14A—C13A—C12A	54.0 (17)
O13B^ii^—Cs1—O14A—Cs2	162.23 (19)	Cs2—O14A—C13A—O13A	95.3 (14)
O13B^ii^—Cs1—O14A—C13A	11.2 (12)	Cs2—O14A—C13A—C12A	−85.0 (13)
O14A^ii^—Cs1—O14A—Cs2	−133.66 (17)	Cs1^vi^—O14B—C13B—O13B	−109.4 (12)
O14A^ii^—Cs1—O14A—C13A	75.3 (12)	Cs1^vi^—O14B—C13B—C12B	71.3 (13)
O13A^iii^—Cs1—O14A—Cs2	−114.2 (5)	Cs2^vi^—O14B—C13B—O13B	138.9 (10)
O13A^iii^—Cs1—O14A—C13A	94.7 (13)	Cs2^vi^—O14B—C13B—C12B	−40.4 (14)
O14B^iii^—Cs1—O14A—Cs2	116.3 (3)	C6A—C1A—C2A—C3A	0.5 (19)
O14B^iii^—Cs1—O14A—C13A	−34.7 (14)	C11A—C1A—C2A—C3A	−179.7 (12)
O13B—Cs1—O13A^i^—Cs2	−2.5 (2)	C2A—C1A—C6A—C5A	−0.2 (18)
O14A—Cs1—O13A^i^—Cs2	−60.15 (19)	C11A—C1A—C6A—C5A	179.9 (12)
O13B—Cs1—O14B^i^—Cs2	49.60 (18)	C2A—C1A—C11A—C12A	167.7 (13)
O14A—Cs1—O14B^i^—Cs2	−7.55 (19)	C6A—C1A—C11A—C12A	−13 (2)
O14A—Cs2—O13B—Cs1	−61.2 (2)	C6B—C1B—C2B—C3B	2.8 (18)
O14A—Cs2—O13B—C13B	63.9 (7)	C11B—C1B—C2B—C3B	−178.7 (11)
O13A^i^—Cs2—O13B—Cs1	−2.5 (2)	C2B—C1B—C6B—C5B	−1.7 (18)
O13A^i^—Cs2—O13B—C13B	122.6 (7)	C11B—C1B—C6B—C5B	179.8 (11)
O14B^i^—Cs2—O13B—Cs1	51.35 (19)	C2B—C1B—C11B—C12B	−164.0 (12)
O14B^i^—Cs2—O13B—C13B	176.5 (8)	C6B—C1B—C11B—C12B	14.4 (19)
O13B^iv^—Cs2—O13B—Cs1	−116.5 (2)	C1A—C2A—C3A—C4A	0 (2)
O13B^iv^—Cs2—O13B—C13B	8.6 (8)	C1B—C2B—C3B—C4B	−3 (2)
O14A^iv^—Cs2—O13B—Cs1	−163.16 (19)	C2A—C3A—C4A—C5A	0 (2)
O14A^iv^—Cs2—O13B—C13B	−38.1 (7)	C2B—C3B—C4B—C5B	2 (2)
O13A^v^—Cs2—O13B—Cs1	129.5 (2)	C3A—C4A—C5A—C6A	0 (2)
O13A^v^—Cs2—O13B—C13B	−105.4 (7)	C3B—C4B—C5B—C6B	−1 (2)
O14B^v^—Cs2—O13B—Cs1	90.3 (5)	C4A—C5A—C6A—C1A	0 (2)
O14B^v^—Cs2—O13B—C13B	−144.6 (7)	C4B—C5B—C6B—C1B	0.7 (19)
O13B—Cs2—O14A—Cs1	59.9 (2)	C1A—C11A—C12A—C13A	−175.6 (12)
O13B—Cs2—O14A—C13A	−142.9 (10)	C1B—C11B—C12B—C13B	−175.3 (11)
O13A^i^—Cs2—O14A—Cs1	−56.3 (2)	C11A—C12A—C13A—O13A	169.0 (13)
O13A^i^—Cs2—O14A—C13A	100.9 (10)	C11A—C12A—C13A—O14A	−10.7 (18)
O14B^i^—Cs2—O14A—Cs1	−7.12 (18)	C11B—C12B—C13B—O13B	1.6 (18)
O14B^i^—Cs2—O14A—C13A	150.1 (9)	C11B—C12B—C13B—O14B	−179.2 (11)

Symmetry codes: (i) *x*, *y*+1, *z*; (ii) −*x*+1, *y*, −*z*+1/2; (iii) −*x*+1, *y*+1, −*z*+1/2; (iv) −*x*, *y*, −*z*+1/2; (v) −*x*, *y*+1, −*z*+1/2; (vi) *x*, *y*−1, *z*.

### Hydrogen-bond geometry (Å, º)

**Table:**

*D*—H···*A*	*D*—H	H···*A*	*D*···*A*	*D*—H···*A*
O14*B*—H14*B*···O14*A*	1.21	1.25	2.462 (10)	180
C11*B*—H11*B*···O13*B*	0.95	2.49	2.830 (14)	101
